# Health-related quality of life and associated factors among epileptic patients on treatment follow up at public hospitals of Wollega zones, Ethiopia, 2018

**DOI:** 10.1186/s13104-019-4720-3

**Published:** 2019-10-22

**Authors:** Muktar Abadiga, Getu Mosisa, Tadele Amente, Adugna Oluma

**Affiliations:** School of Nursing and Midwifery, Institute of Health Sciences, Wollega University, Nekemte, Ethiopia

**Keywords:** Epilepsy, Quality of life, Wollega zones

## Abstract

**Objective:**

The aim of this study was to assess health-related quality of life and its associated factors among epileptic patients in public hospitals of Wollega zones, Ethiopia. Institutional based cross-sectional study was conducted on 402 epileptic patients, from March 01 to March 30, 2018. Multiple linear regression with backward elimination was used, and all analyses were conducted at the 0.05 significance level.

**Results:**

The overall mean total score on the WHOQOL-BREF scale was 60.47 with ± 23.07 SD. Monthly income ≤ 500 EB (β = − 12.49, P < 0.001), living alone (β = − 7.11, P = 0.007), adverse drug reaction (β = − 10.86, P < 0.001), comorbidity of anxiety (β = − 12.99, P < 0.001), perceived social stigma (β = − 9.73, P < 0.001) and frequency of seizure once per week (β = − 8.41, P = 0.001) were negatively associated with quality of life of epileptic patients. The mean quality of life of patients living with epilepsy in this study was low. The clinician should early recognize and treat drug side effects, detect and manage comorbidity, and control seizure in order to increase quality of life of epileptic patients.

## Introduction

Epilepsy is a chronic brain disorder characterized by recurrent seizures [1]. It affects about 50 million people worldwide, and 85% of them live in developing countries. Its annual incidence is about 50 cases per 100,000 persons in developed countries and 100–190 cases per 100,000 persons in developing countries [2]. The psychosocial effects of epilepsy have led to the need to quantify quality of life in epileptic patients [3]. Quality of life is defined as an individual’s perception of their position in life in the context of the culture and value systems in which they live [4]. Quality of life is worse in epileptic patients than in the general population [5]. A cross-sectional comparative study done in Kenya showed the mean quality of life among people living with epilepsy (49.90%) was significantly (p < 0.01) lower than that of the normal controls (77.60%) accompanying them and significantly impaired as compared to the hypothesized mean of 75 ± 2.5% [6]. In Ethiopia, 45.8% of epileptic patients have a poor quality of life [7].

Unpredictable progression of the disease, stigma, poor self-esteem, anxiety and depression, unemployment, social isolation, and cognitive problems has been reported to impair quality of life in epileptic patients [8]. Physical injuries such as burns, drowning, falls, and car accidents can endanger the lives of epileptic patients [9]. The patients may isolate themselves from society because people most often stigmatize them. As a result, they can suffer from depression which may further decrease their quality of life [10, 11].

Apart from the disease, the antiepileptic medications can be a burden on the patients. Their side effects such as fatigue, memory problem, difficulty concentrating, and drowsiness, difficulty in thinking clearly, and nervousness or agitation affects their quality of life [12]. Their side effects, especially for poly-therapies, have negatively associated with health-related quality of life, independent of seizure frequency [13–16].

However; in Africa and particularly in Ethiopia, few studies have been conducted on the health-related quality of life of epileptic patients [17]. Therefore; this study was aimed to identify factors influencing health-related quality of life of patients with epilepsy in public hospitals of Wollega zones.

## Main text

### Methods

#### Study setting and population

This study was conducted in six randomly selected hospitals of Wollega zones from March 01 to March 30, 2018. The institutional based cross-sectional study design was employed. All epileptic patients on treatment follow up at selected public hospitals of Wollega zones was the source population and the sampled epileptic patients was the study population. All patients whose ages were 18 years and above were included in the study and persons who are eligible but not willing to take part in the study were excluded from the study.

#### Sample size determination and sampling techniques

The sample size of the study was determined using the formula for estimation of a single population proportion. 45.8% (0.458) proportion of population living with epilepsy who had poor QOL was taken from similar a study done in Addis Ababa [7], and by adding a non-response rate of 5%, a total of 402 epileptic patients were involved in the study. Simple random sampling method was used to select the study participants.

#### Data collection tool and procedures

Data was collected using a structured questionnaire. Quality of Life questionnaire (WHOQOL BREF) scale, Hospital Anxiety and Depression scale (HADS) and stigma scale were used, and face to face interview was employed. WHOQOL-BREF contains 26 items consisting four domains were used: physical health, psychological health, social relationships, and environmental health. Each item of the WHOQOL-BREF was scored from 1 (very dissatisfied/very poor) to 5 (very satisfied/very good). Each questionnaire is scored on a scale of 24 to 120, with a higher WHOQOL score indicating better quality of life.

#### Data processing and analysis

The data were coded, cleaned and entered into Epi data version 3.1 and exported into SPSS window version 20.0 for analysis. Descriptive statistics were expressed in frequency, percentage, mean and standard deviation. Simple linear regression was used to find an association between dependent variable and each independent variable. Multiple linear regression with backward elimination was used to identify independent factors which best predict quality of life. All analyses were conducted at the 0.05 significance level.

#### Data quality control

The questionnaire was translated to the local language and then translated back to English by expertise to check for consistency. Five percent of the questionnaire was pre-tested on epileptic patients at the same study area 5 days before data collection, and some modification of the questionnaire were made. One-day training was given for data collectors and supervisors on how to collect and handle the data.

## Results

### Socio-demographic characteristics of the respondents

Of the total of 402 study participants sampled, 392 were participated; with a response rate of 97.5%. From the total of 392 participants, 203 (51.8%) were male, and 189 (48.2%) were females. The mean age of the study participant was 30.7 years with ± 10.04 standard deviation. Majority of the study participant was unmarried, 226 (57.7%) followed by married, which accounts 145 (37.0%). Concerning educational status, 108 (27.6%) were completed grade 9–10, 105 (26.8%) were completed grade 1–8 and 71 (18.1%) have degree and above. Regarding monthly income, 124 (31.6%) gets monthly income 501–1000 EB followed by monthly income greater than 2000 EB which accounts 99 (25.3%); whereas the mean monthly income was 1490.72 ± 1183.5 SD. Concerning living condition, 333 (84.9%) live with parents (Table 1).

**Table 1:** Distribution of participants by socio-demographic and clinical characteristics at public hospitals of Wollega zone, 2018 (n = 392)

Variables	Frequency	Percentage
Gender		
Female	189	48.2
Male	203	51.8
Ethnicity		
Oromo	320	81.6
Amhara	54	13.8
Gurage	5	1.3
Tigre	4	1.0
Others	9	2.3
Total	392	100
Age		
18–24	125	31.9
25–34	147	37.5
35–44	68	17.3
≥ 45	52	13.3
Total	392	100
Religion		
Muslim	42	10.7
Orthodox	89	22.7
Protestant	226	57.7
Catholic	27	6.9
Others	8	2.0
Total	392	100
Marital status		
Married	145	37.0
Unmarried	226	57.7
Widowed	11	2.0
Divorced	10	2.6
Total	392	100
Educational status		
No formal education	44	11.2
Grade 1–8	105	26.8
Grade 9–10	108	27.6
Diploma	64	16.3
Degree and above	71	18.1
Total	392	100
Occupational status		
Gov’t employee	68	17.3
Student	61	15.6
Farmer	147	37.5
Merchant	69	17.6
Daily laborers	36	9.2
Others	11	2.8
Total	392	100
Residence		
Urban	66	16.8
Rural	326	83. 2
Total	392	100
Monthly income		
≤ 500	67	17.1
501–1000	124	31.6
1001–1500	68	17.3
1501–2000	34	8.7
≥ 2001	99	25.3
Total	392	100
Duration of disease (years)		
< 2	25	6.4
2–5	106	27.0
6–10	113	28.8
> 10	148	37.8
Total	392	100
Type of epilepsy		
Generalized	193	49.2
Focal	199	50.8
Total	392	100
Type of therapy		
Monotherapy	260	66.3
Polytherapy	132	33.7
Total	392	100
Adverse drug reaction		
Yes	182	46.4
No	210	53.6
Total	392	100
Co-morbidity of anxiety		
Yes	116	29.6
No	276	70.4
Total	392	100
Co-morbidity of depression		
Yes	46	11.7
No	346	88.3
Total	392	100
Perceived social stigma		
Yes	132	33.7
No	260	66.3
Total	392	100
Frequency of seizure		
One per week	163	41.6
One per month	133	33.9
One per year	60	15.3
None in past year	36	9.2
Total	392	100

### Clinical characteristics of the respondents

Concerning the disease duration, 148 (37.8%) had duration of > 10 years, followed by 6–10 years (28.8%). Regarding the type of epilepsy, almost half of the study participants had a focal type which is 199 (50.8%). Majority of the study participants, 210 (53.6%) had no adverse drug reaction, and 182 (46.4%) had experienced an adverse drug reaction. Regarding co-morbidity, 116 (29.6%) had anxiety, and 46 (11.7%) had depression. One hundred thirty-two, (33.7%) had perceived stigma, and 313 (79.8%) had a fear of having a seizure. Out of the total respondents, 163 (41.6%) had one seizure attacks per week followed by one seizure attacks per month, 133 (33.9%) (Table 1).

### Whoqol-bref (0–100) characteristics of respondents

Descriptive statistics were performed to find out means and standard deviations of WHOQOL measurement scale. The WHOQOL BREF covers four different domains of quality of life, physical, psychological, social and environmental. The mean score for physical dimension is 17.89 ± 6.5 SD with a range of 8 to 32. The mean score for the Psychological dimension is 15.33 ± 6.17 SD with a range of 7 to 27. The mean score of social dimensions is 7.69 ± 3.24 SD with a range of 3 to 12. The mean score for environmental dimension is 19.56 ± 7.23 SD with a range of 9 to 34. The overall mean total score on the WHOQOL-BREF scale is 60.47 with ± 23.14 standard deviation. The minimum score of this WHOQOL BREF in this study is 27.00 and the maximum score is 105.00 with the range of score 78.00 (Additional file 1: Table S1).

### Bivariable linear regression analysis

Simple linear regression analysis was done to investigate how much each independent variable were associated with quality of life. In simple linear regression analysis, socio-demographic variables such as marital status, educational status, occupation, residence, living condition and monthly income showed significant association with quality of life. Clinical variables such as duration of disease, adverse drug reaction, co-morbidity of anxiety, comorbidity of depression, perceived social stigma and frequency of seizure were also showed significant association with health-related quality of life (Table 2).

**Table 2:** Simple linear regression analysis of factors associated with Quality of life among epileptic patients attending public hospitals of Wollega zones, West Ethiopia, 2018

Variables	Unstandardized coefficient		Standardized coefficient	t value	P-value
	Beta	Standard error	Beta		
Gender					
Female vs male	− 2.49	2.33	− 0.05	− 1.07	0.28
Ethnicity					
Amhara vs Oromo	2.40	10.42	0.01	0.23	0.81
Gurage vs Oromo	− 6.8	11.63	− 0.03	− 0.58	0.55
Tigre vs Oromo	− 3.59	7.80	− 0.02	− 0.46	0.64
Religion					
Muslim vs protestant	− 0.27	3.71	− 0.005	− 0.07	0.94
Orthodox vs protestant	1.24	3.18	0.02	0.39	0.69
Catholic vs protestant	4.40	8.64	0.02	0.50	0.61
Educational status					
No formal education vs ≥ degree	− 8.66	3.07	− 0.16	− 2.81	0.005
Grade 1–8 vs ≥ degree	− 10.39	3.05	− 0.20	− 3.40	0.001
Grade 9–10 vs ≥ degree	− 7.87	3.55	− 0.12	− 2.21	0.027
Occupation					
Student vs Gov’t employee	− 10.12	2.72	− 0.21	− 3.71	< 0.001
Farmer vs Gov’t employee	− 3.91	3.37	− 0.06	− 1.15	0.24
Merchant vs Gov’t employee	− 14.30	4.26	− 0.17	− 3.35	0.001
Daily laborers vs Gov’t employee	− 1.21	7.10	− 0.009	− 0.17	0.86
Marital status					
Unmarried vs married	− 11.59	7.00	− 0.08	− 1.70	0.08
Widowed vs married	− 16.73	7.34	− 0.11	− 2.27	0.02
Residence					
Urban vs rural	8.70	3.08	0.14	2.82	0.005
Monthly income					
≤ 500 vs > 2000 EB	− 16.81	2.60	− 0.33	− 6.44	< 0.001
501–1000 vs > 2000 EB	− 10.12	3.16	− 0.16	− 3.20	0.001
1001–1500 vs > 2000 EB	− 10.58	4.13	− 0.12	− 2.55	0.011
Living condition					
Alone vs with parents	− 15.65	3.16	− 0.24	− 4.94	< 0.001
Type of epilepsy					
Generalized vs focal	0.65	2.33	0.01	0.28	0.77
Adverse drug reaction					
Yes vs no	− 18.49	2.14	− 0.40	− 8.62	< 0.001
Co-morbidity of anxiety					
Yes vs no	− 21.02	2.32	− 0.41	− 9.04	< 0.001
Co-morbidity of depression					
Yes vs no	− 22.35	3.44	− 0.31	− 6.48	< 0.001
Fear of having seizure					
Yes vs no	− 4.78	2.89	− 0.08	− 1.65	0.100
Perceived social stigma					
Yes vs no	− 13.04	2.38	− 0.26	− 5.48	< 0.001
Age at onset of disease					
10–19 years vs < 10 years	0.13	2.87	0.002	0.04	0.96
20–29 years vs < 10 years	7.40	3.95	0.09	1.87	0.06
≥ 40 years vs < 10 years	2.33	9.52	0.01	0.24	0.80
Frequency of seizure					
One per year vs none in past year	− 8.34	2.53	− 0.17	− 3.29	0.001
One per month vs none in past year	− 19.20	3.27	− 0.30	− 5.82	< 0.001
One per week vs none in past year	− 23.11	3.98	− 0.29	− 5.79	< 0.001

Predictor variables: Sociodemographic and clinical variables. Dependent variables: health related quality of life

### Multivariable linear regression analysis

Multivariable linear regression was conducted to examine the best combination of factors for predicting quality of life. So, multivariable linear regressions model with backward elimination was used to extract factors that best predict quality of life. In the final model of multivariable linear regression, monthly income, living condition, adverse drug reaction, co-morbidity of anxiety, perceived social stigma and frequency of seizures were significantly associated with health-related quality of life. Multivariable linear regression analyses showed that monthly income ≤ 500 EB, 501–1000 EB and 1001–1500 EB were negatively associated with health-related quality of life (β = − 12.49, P < 0.001, β = − 10.10, P < 0.001 and β = − 8.41, P = 0.012 respectively) when compared with monthly income > 2000 EB. The finding also showed that living alone were negatively associated with health-related quality of life (β = − 7.11, P = 0.007) when compared with those who live with their parents.

Presence of adverse drug reaction was negatively associated with health-related quality of life (β = − 10.86, P < 0.001). The result also showed that comorbidity of anxiety was negatively influenced health related quality of life (β = − 12.99, P < 0.001). Presence of social stigma was negatively associated with health-related quality of life (β = − 9.73, P < 0.001), and the frequency of seizure once per week showed negative association with health-related quality of life of epileptic patients (β = − 8.4, P = 0.001). In the multivariable regression, the model is significant (F = 20.42, *P* value < 0.001) and has an adjusted R^2^ = 0.458. The adjusted R^2^ of 0.458 (45.8%) of this model indicates that the variables included in the model explain about 45.8% of the variance or change in quality of life (Table 3).

**Table 3:** Ordinary least square regression analysis of factors associated with quality of life among epileptic patients attending public hospitals of Wollega zones, West Ethiopia, 2018

Variables	Unstandardized coefficient		Standardized coefficient	t value	P-value	Multicollinearity statistics	
	Beta	Standard error	Beta			Tolerance	VIF
Monthly income							
≤ 500 vs > 2000 EB	− 12.49	2.19	− 0.25	− 5.70	< 0.001	0.71	1.40
501–1000 vs > 2000 EB	− 10.10	2.63	− 0.16	− 3.83	< 0.001	0.74	1.34
1001–1500 vs > 2000 EB	− 8.41	3.33	− 0.10	− 2.52	0.012	0.83	1.19
Living condition							
Alone vs with parents	− 7.11	2.63	− 0.11	− 2.70	0.007	0.83	1.20
Adverse drug reaction							
Yes vs no	− 10.86	1.83	− 0.23	− 5.92	< 0.001	0.88	1.13
Co-morbidity of anxiety							
Yes vs no	− 12.99	2.03	− 0.25	− 6.39	< 0.001	0.85	1.16
Perceived social stigma							
Yes vs no	− 9.73	1.94	− 0.20	− 5.00	< 0.001	0.87	1.14
Frequency of seizure							
One per week vs none in past year	− 8.41	2.52	− 0.13	− 3.33	0.001	0.89	1.12

Intercept (constant) = 42.15; standard error = 3.62; t-value = 11.63; sig. < 0.001; adjusted R square = 0.458 (45.8%), F = 20.42, P < 0.001. Dependent variables: health related quality of life

## Discussion

In this study, we estimated the quality of life of peoples living with epilepsy. The result showed that the mean quality of life is 60.47 ± 23.07 SD. The mean quality of life of in this study is similar with a study done in Uganda [18] and Addis Ababa [7]. However; the mean quality of life in the present study is lower than a study done in India [19] and Malaysia [24]. On the other hand, the mean quality of life of this study is higher than the study done in Russia [20] and Australia [25]. The possible reason for the discrepancy might be due to inadequate sample size and non-random sampling techniques in Russian study. The other possible reason could be this cross-sectional study was conducted using WHOQOL-BREF, but others were conducted using the Quality of Life Inventory for Epilepsy (QOLIE-31).

In this study, respondent’s monthly income was significantly associated with health-related quality of life, which is in line with a study done in Kenya [6] and in Addis Ababa at Amanuel Mental Specialized Hospital [7]. The finding of this study showed that adverse drug reaction was associated with quality of life of epileptic patients. This finding is consistent with a study done in Uganda [18], India [19] and Russia [20]. The finding of this study also showed that co-morbidity of anxiety was associated with quality of life of epileptic patients which is supported by a study done in Addis Ababa [7].

Moreover, in this study, most of the respondents who had frequent seizures reported the poor quality of life. This finding is consistent with many previous studies conducted in different parts of the world [7, 18–23]. This finding is also consistent with a study done in Kenya [6] and Malaysia [24] and Australia [25]. The results of the present study also revealed that the HRQOL is significantly influenced by stigmatization about their disease, which is similar with a study done in Addis Ababa [7] and Australia [25]. Unlike to other studies, age, marital status, educational level and gender were not significantly associated with health-related quality of life.

## Conclusion

The mean health-related quality of life of people living with epilepsy in this study was low. Respondent’s monthly income, living condition, adverse effects of antiepileptic medications, co-morbidity of anxiety, perceived stigma, and frequency of seizure have significantly affected the quality of life of epileptic patients. The clinician should early recognize and treat drug side effects, early detect and manage comorbidity, and control seizure in order to increase quality of life of epileptic patients.

## Limitation of the study

The research design is cross-sectional in nature and cannot confirm causality.

## Supplementary information

**Additional file 1: Table S1.** Means and standard deviation of overall HRQOL and its subscale/dimension scores among epileptic patients attending public hospitals of Wollega Zones, West Ethiopia, 2018.

## Data Availability

The data used during the current study are available from the corresponding author on request.

## Abbreviations

ADRs: adverse drug reactions
AEDs: anti-epileptic drugs
HADS: Hospital Anxiety and Depression Scales
HRQOL: health related quality of life
QOL: quality of life
QOLIE: quality of life in epilepsy inventory
SPSS: Statistical Package for Social Science
PWE: people with epilepsy
WHO: World Health Organization
WHOQOL: World Health Organization Quality Of Life
