# Supplementary material for: Health-related quality of life and associated factors among epileptic patients on treatment follow up at public hospitals of Wollega zones, Ethiopia, 2018
Source: BMC Res Notes. 2019 Oct 22;12:679. doi: 10.1186/s13104-019-4720-3 (PMC6805515; doi:10.1186/s13104-019-4720-3)
Supplement: Supplementary file 1 — Additional file 1: Table S1. Means and standard deviation of overall HRQOL and its subscale/dimension scores among epileptic patients attending public hospitals of Wollega Zones, West Ethiopia, 2018. [file 13104_2019_4720_MOESM1_ESM.docx]

**S1 table: Means and standard deviation of overall HRQOL and its subscale/dimension scores among epileptic patients attending public hospitals of Wollega Zones, West Ethiopia, 2018.**

| **HRQOL dimension** | **Mean** | **SD** | **Median** | **Min. score** | **Max. score** |
| --- | --- | --- | --- | --- | --- |
| Physical dimension | 17.89 | 6.50 | 18.00 | 8.00 | 32.00 |
| Psychological dimension | 15.33 | 6.17 | 15.50 | 7.00 | 27.00 |
| Social dimension | 7.69 | 3.24 | 7.50 | 3.00 | 12.00 |
| Environmental dimension | 19.56 | 7.23 | 19.00 | 9.00 | 34.00 |
| Overall HRQOL score | 60.47 | 23.14 | 60.00 | 27 | 105 |
